# Management of Coronary Artery Calcium and Coronary CTA Findings

**DOI:** 10.1007/s12410-015-9334-0

**Published:** 2015-04-15

**Authors:** Dustin M. Thomas, Sanjay Divakaran, Todd C. Villines, Khurram Nasir, Nishant R. Shah, Ahmad M. Slim, Ron Blankstein, Michael K. Cheezum

**Affiliations:** Department of Medicine (Cardiology Service), San Antonio Military Medical Center, San Antonio, TX USA; Department of Medicine, Brigham and Women’s Hospital and Harvard Medical School, Boston, MA USA; Department of Medicine (Cardiology Service), Walter Reed National Military Medical Center, Bethesda, MD USA; Center for Prevention and Wellness Research, Baptist Health Medical Group, Miami Beach, FL USA; Noninvasive Cardiovascular Imaging Program, Departments of Medicine (Cardiovascular Division) and Radiology, Brigham and Women’s Hospital, Boston, MA USA; Non-invasive Cardiovascular Imaging Program, Brigham and Women’s Hospital, 75 Francis St, Boston, MA 02115 USA

**Keywords:** Coronary CTA, Coronary artery calcium, Management, Prognosis, Chest pain, Cardiovascular disease, Acute coronary syndrome, Ischemic heart disease, Major adverse cardiac events, High risk plaque, atherosclerosis

## Abstract

Coronary artery calcium (CAC) testing and coronary computed tomography angiography (CTA) have significant data supporting their ability to identify coronary artery disease (CAD) and classify patient risk for atherosclerotic cardiovascular disease (ASCVD). Evidence regarding CAC use for screening has established an excellent prognosis in patients with no detectable CAC, and the ability to risk re-classify the majority of asymptomatic patients considered intermediate risk by traditional risk scores. While data regarding the ideal management of CAC findings are limited, evidence supports statin consideration in patients with CAC > 0 and individualized aspirin therapy accounting for CAD risk factors, CAC severity, and factors which increase a patient’s risk of bleeding. In patients with stable or acute symptoms undergoing coronary CTA, a normal CTA predicts excellent prognosis, allowing reassurance and disposition without further testing. When CTA identifies nonobstructive CAD (<50 % stenosis), observational data support consideration of statin use/intensification in patients with extensive plaque (at least four coronary segments involved) and patients with high-risk plaque features. In patients with both nonobstructive and obstructive CAD, multiple studies have now demonstrated an ability of CTA to guide management and improve CAD risk factor control. Still, significant under-treatment of cardiovascular risk factors and high-risk image findings remain, among concerns that CTA may increase invasive angiography and revascularization. To fully realize the impact of atherosclerosis imaging for ASCVD prevention, patient engagement in lifestyle changes and the modification of ASCVD risk factors remain the foundation of care. This review provides an overview of available data and recommendations in the management of CAC and CTA findings.

## Introduction

Heart disease continues to be the most common cause of death in the United States, despite a downward trend in mortality due to heart disease since the late 1960s [1, 2]. This decrease in mortality is likely owing to multiple factors, including improved medical therapies, improved management of acute coronary syndromes (ACS), and a decreased prevalence of smoking [3–6]. Despite this progress, the prevalence of heart disease, coronary heart disease, myocardial infarction (MI), and stroke has not been appreciably affected since the late-1990s [7].

Currently, the standard evaluation of asymptomatic patients at risk for major adverse cardiac events (MACE) relies on a variety of risk calculators to guide management. Unfortunately, risk scores are known to significantly misclassify risk, particularly among women and younger individuals [8]. Conversely, patients with symptoms concerning for ischemic heart disease typically undergo stress testing with or without imaging to stratify risk and guide management. While noninvasive functional tests offer improved specificity to detect ischemia, these techniques have limited sensitivity to detect subclinical coronary artery disease (CAD) [9••]. Thus, a more sensitive test to detect CAD may enhance risk assessment and improve patient management beyond modern practice. This review will focus on the use of coronary artery calcium (CAC) testing and coronary computed tomographic angiography (CTA) to detect CAD and risk stratify patients, with implications of these tests on patient management.

## Coronary Artery Calcium Testing

CAC testing requires no patient preparation, avoids the use of intravenous contrast, and can be obtained in any patient able to lay flat and perform a single breath hold. The amount of CAC is most commonly quantified by the Agatston method—accounting for the area of calcified plaque and the corresponding computed tomography (CT) density [10]. This score may be compared with a database from the Multi-ethnic Study of Atherosclerosis (MESA) to provide a percentile of CAC severity among age, gender, and ethnicity-matched peers [11].

### Limitations

CAC testing exposes the patient to a low dose of radiation (∼1 mSv), akin to mammography and less than annual exposure from natural sources (∼3-4 mSv). By comparison, a position statement from the Health Physics Society argues that below 50–100 mSv, “the risks of health effects from radiation are either too small to be observed or are nonexistent” [12]. Consequently, recommendations from the European Society of Cardiac Radiology and the North American Society for Cardiovascular Imaging argue that CAC testing is unlikely to carry additional risk beyond natural background radiation exposure, and should not be avoided when the results of testing have the potential to meaningfully impact therapy [13]. Additionally, CAC scanning may detect incidental extra-cardiac findings such as pulmonary nodules, with a low percentage (∼10 %) of these findings requiring further workup [14].

### CAC Risk Classification and the Potential to Improve Outcomes

To date, CAC screening has been studied in more than 100,000 patients, including multiple large prospective studies with up to 10-year follow-up [15, 16]. Across these studies, CAC has demonstrated a consistent ability to assess cardiovascular risk in asymptomatic patients beyond that provided by alternative noninvasive tests and risk calculators. The utility of CAC screening is most evident among patients categorized as intermediate risk by traditional measures. In the MESA and Heinz-Nixdorf Recall studies, for example, net reclassification improvement was achieved in 54.4 % and 65.6 % of patients deemed ‘intermediate’ risk by standard assessment, respectively [17]. Thus, CAC appropriately reclassified a majority of patients into low- and high-risk categories—an important distinction when counseling patients and directing therapies. Additionally, data from MESA highlight the discordance of risk factors and CAC severity, where CAC = 0 in 35 % of patients with ≥3 modifiable risk factors, compared with 12 % and 5 % of patients with no risk factors having CAC >100 and >300, respectively [18]. Thus, even patients with low and high risk by traditional risk assessment tools may benefit from CAC testing. Across studies, patients with CAC = 0 have a very low risk of future events (<0.1 %/year risk of coronary heart disease [CHD]) [19]. Conversely, patients with CAC ≥ 100 are at significant risk of CHD with a tenfold increased event rate compared with CAC = 0 [19]. Even minimal CAC (CAC = 1–10) carries a twofold increase in CV events (stroke, myocardial infarction, or cardiac death) over 10 years compared with CAC = 0 [20].

While the benefits of CAC testing are debated among strategies to guide cardiovascular risk assessment, appropriate use criteria [21, 22••] and guidelines [23, 24•, 25•] provide variable support for CAC testing (Table 1) [26]. The 2013 American College of Cardiology/American Heart Association (ACC/AHA) guideline on cardiovascular risk assessment recommend a IIb indication for CAC testing in selected patients while expanding indications for statin therapy utilizing a new pooled cohort equation and four patient categories most likely to benefit from treatment (Table 1, legend) [25•]. By increasing the number of patients eligible for statins, this approach improves overall sensitivity to identify patients with CAD who benefit from treatment, at the expense of reduced specificity [27]. Notably, the pooled cohort equation was derived from studies examining a combined outcome of atherosclerotic cardiovascular disease (ASCVD) including stroke. However, the majority of strokes (∼60 %) worldwide are of cardioembolic, lacunar, or hemorrhagic origin and thus are less likely to benefit from statins [28]. In addition, recent evidence suggests that risk calculators may significantly overestimate ASCVD risk [29].

**Table 1:** Recommendations for CAC testing in asymptomatic patients

Appropriate use criteria				
	Low-risk	Low-risk + family history early CAD^a^	Intermediate risk	High-risk
2010 SCCT/ACC/AHA appropriate use criteria [22••]	Inappropriate	Appropriate	Appropriate	Uncertain
2014 ACR appropriate use criteria [21]	Usually not appropriate	May be appropriate	Appropriate	Usually not appropriate
Guideline statements				
2010 ACC/AHA Guidelines [24•]	IIb indication: low–intermediate risk (6–10 % 10-year risk)			
	IIa indication: intermediate risk (10–20 % 10-year risk)			
2012 ESC guidelines [23]	IIa indication: “[CAC] should be considered for cardiovascular risk assessment in asymptomatic adults at moderate risk”			
2013 ACC/AHA guidelines [25•]	IIb indication: “If, after quantitative risk assessment, a risk-based treatment decision is uncertain, assessment [of CAC] may be considered to inform treatment decision making.”^b^			

*ACC* American College of Cardiology, *ACR* American College of Radiology, *AHA* American Heart Association, *CAC* coronary artery calcium, *CAD* coronary artery disease, *ESC* European Society of Cardiology, *SCCT* Society of Cardiovascular Computed Tomography

^a^First-degree relative male < 55 years of age or female < 65 years of age

^b^After discussion with patient, when decision to initiate statin therapy is unclear among selected individuals who are not in one of the four statin benefit groups, defined as those with (i) clinical atherosclerotic cardiovascular disease (ASCVD), (ii) primary elevation of low-density lipoprotein cholesterol (LDL-C) ≥ 190 mg/dL, (iii) age 40–75 years with diabetes and LDL-C 70-189 mg/dL, or (iv) age 40–75 years without clinical ASCVD or diabetes and LDL-C 70–189 mg/dL and estimated 10-year ASCVD risk ≥ 7.5 %. Adapted with permission from Divakaran et al. [26]

By comparison, the 2010 ACC/AHA guidelines for cardiovascular risk assessment provided a IIa recommendation for CAC testing among intermediate risk patients considering prognostic evidence and early studies examining the potential for CAC to guide management and improve outcomes [24•]. In the single-center St. Francis study, 1,007 patients with CAC > 80th percentile for age and sex were randomized to placebo versus low-dose atorvastatin 20 mg daily [30]. While the primary outcome of major cardiovascular events failed to reach statistical significance, a strong trend towards fewer events was noted in the atorvastatin group (6.9 % vs. 9.9 %, *p* = 0.08). Additionally, in the subset of patients with CAC > 400, there was a 42 % reduction in relative risk and a 6.3 % absolute risk reduction with atorvastatin compared with placebo (*p* < 0.05). In another randomized trial, the EISNER study (Early Identification of Subclinical Atherosclerosis by Noninvasive Imaging Research) examined 2,137 patients randomized to CAC versus no CAC testing [31]. Though this study lacked hard outcome data, patients with CAC testing had significant improvements in blood pressure (*p* = 0.02), cholesterol levels (*p* = 0.04), waist circumference (*p* = 0.01), and Framingham risk score (*p* = 0.003) compared with patients without CAC testing at 4-year follow-up. Notably, downstream testing and costs were similar between CAC and no CAC groups, balanced by lower and higher resource utilization in patients with CAC = 0 and CAC ≥ 400, respectively.

### Management of CAC Findings

When the decision is made to perform CAC testing, management of CAC findings requires shared decision making with the patient considering risks and benefits of therapies, patient preference, and tolerance to long-term medication use.

#### Recommendations for Aspirin Use

**Fig. 1:**
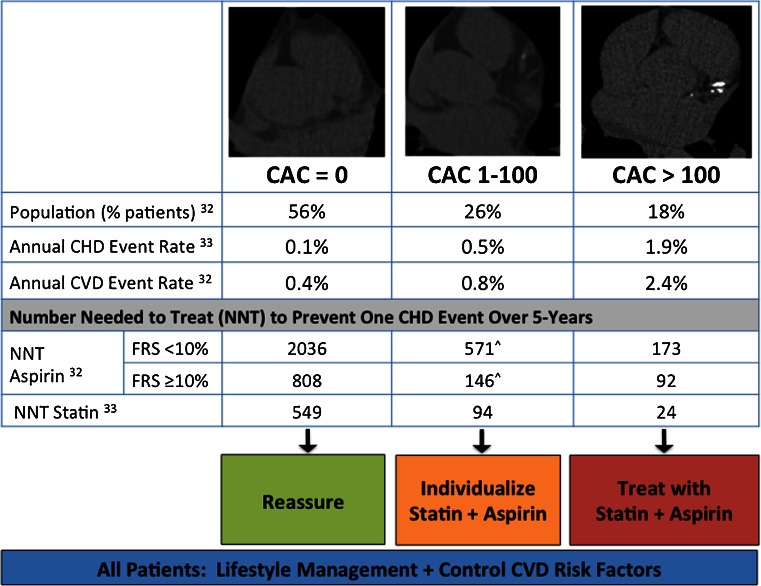
CAC score prognosis and recommended treatment strategy. ^Note that the estimated number needed to harm with aspirin use is 442 patients to cause one major bleeding episode over a 5-year period [32]. Thus, consider aspirin use in patients with CAC 1-100 when anticipated benefit exceeds risk (e.g., when FRS ≥ 10 %). *CAC*, coronary artery calcium (Agatston units); *CHD*, coronary heart disease; *CVD*, cardiovascular disease; *FRS*, Framingham risk score; *NNT*, number needed to treat. Adapted with permission from Divakaran et al. [26]

Available data do not support therapy with aspirin for those with a CAC score of zero in whom the risks of treatment are likely to outweigh benefits of therapy. In data from Miedema et al. examining the risk/benefits of aspirin use from MESA, the number needed to harm by 5-year aspirin use was approximately 442 treated patients to cause one major bleeding episode [32]. Conversely, the number needed to treat (NNT) with aspirin to prevent one major cardiac event over the same time period in patients with a CAC of 0 and Framingham Risk Score (FRS) ≥ 10 % was NNT = 808 [32]. These data suggest that patients with a CAC score of zero, regardless of FRS, should not be routinely treated with aspirin. In contrast, aspirin therapy should be strongly considered in patients with a CAC score >100 who derive the most benefit from aspirin use (Fig. 1) [17, 26, 32, 33]. While patients with a CAC score of 1–100 are at increased risk for MACE relative to no CAC, the risk of major bleeding from aspirin in this group appears to outweigh benefits when FRS is low (<10 %) (Fig. 1). In the majority of patients at low risk for cardiovascular disease (CVD), it is important to recognize that the benefits of aspirin use for primary prevention remain in doubt, with variable recommendations for aspirin therapy among guidelines [34]. In the 2009 Antithrombotic Trialists’ Collaboration, aspirin provided no benefit in the combined outcome of cardiovascular death compared with no aspirin, incorporating data from six major trials of 95,000 individuals who were generally low risk for CVD [35••]. While aspirin provided low absolute risk reductions [ARR] in death from coronary heart disease (ARR 0.06 %/year), nonfatal MI (ARR 0.05 %/year), and ischemic stroke (ARR 0.01 %/year) versus no aspirin, these gains were offset by an increased risk of major extracranial bleeding and hemorrhagic stroke on aspirin (0.3 %/year and 0.1 %/year, respectively).

Consequently, aspirin use in patients with a CAC score of 1–100 requires individualization of therapy incorporating CVD risk (e.g., consider when FRS ≥ 10 %) and patient factors that may increase the risk of bleeding (e.g., history of gastrointestinal bleeding or hemorrhagic stroke, age > 65, alcohol use, liver/renal disease, or concomitant use of non-steroid anti-inflammatory drugs, anticoagulant, or antiplatelet agents).

#### Recommendations for Statin Use

Relative to aspirin therapy, statin use generally confers less risk with greater benefit by a comparison of NNT to prevent one CHD-event over 5 years (Fig. 1). Consequently, statin therapy should be considered in all patients with a CAC score > 0 [26]. Exception in statin use should be noted among patients with CAC = 0, where attention may focus on cardiovascular health metrics known to improve cardiovascular morbidity and mortality [36]. Currently, the role of statin therapy in patients with CAC = 0 and patients at high risk by standard scoring methods (e.g., Framingham risk > 20 %) remains unclear and is a focus of ongoing study.

## Coronary CTA

**Fig. 2:**
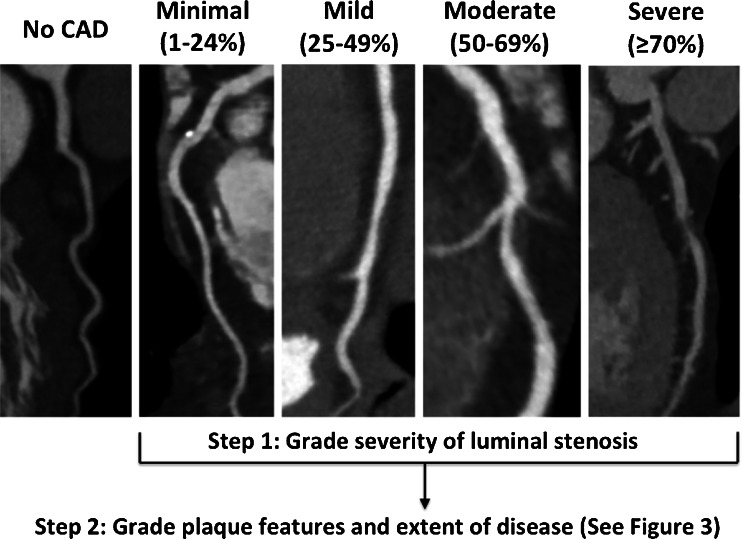
Coronary CTA identified CAD severity. Recommended quantitative stenosis grading of CAD assessed by coronary CTA [40]. Following stenosis assessment, recommend grading of CTA-identified plaque features and CAD extent (see Fig. 3). *CAD*, coronary artery disease; *CTA*, computed tomographic angiography

Coronary CTA has emerged as a powerful tool for the diagnosis of CAD and has been shown in multiple large trials to exclude obstructive CAD with a high negative predictive value (>95 %) [37]. In practice, CTA relies on adequate patient preparation including heart rate control, administration of intravenous contrast, and utilization of high-resolution (≥64-slice) CT to optimally assess coronary plaque. To guide the use of CTA, consensus statements are available regarding patient selection [22••], CTA performance [38], and user training [39]. Additionally, CTA guidelines offer recommendations for the reporting of CAD severity (Fig. 2) to ensure consistent and effective communication with referring physicians [40].

**Fig. 3:**
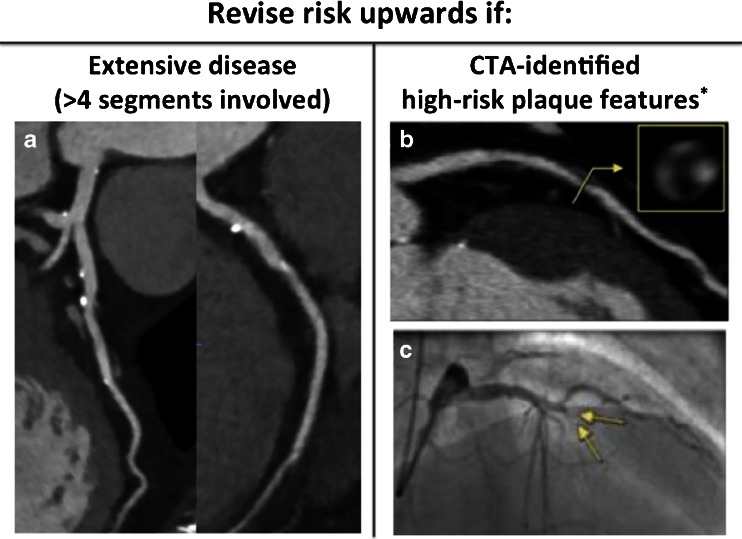
Coronary CTA features associated with an increased risk of major adverse cardiac events. **a** Extensive nonobstructive calcified plaque in the left main, proximal-mid LAD, proximal left circumflex, and first obtuse marginal arteries. **b** An atherosclerotic plaque with positive remodeling, low-attenuation plaque, and napkin-ring sign in the proximal LAD on coronary CTA*. **c** Invasive coronary angiography demonstrating occlusion of the proximal LAD at the site of high-risk plaque 10 months after coronary CTA (*arrows*). *Reproduced with permission from Otsuka et al. [41]. *CTA*, computed tomographic angiography; *LAD*, left anterior descending

Beyond luminal stenosis, CTA provides an assessment of plaque composition (calcified/noncalcified), akin to a combination of intravascular ultrasound and invasive coronary angiography (ICA) [42]. To quantify plaque, multiple scoring methods are now available to grade CAD severity and/or extent, including the segment involvement score (SIS), a measure of the total number of coronary segments with any atherosclerosis (Fig. 3a). By measuring the total plaque burden, CTA provides noninvasive measurement of the full spectrum of CAD to include subclinical atherosclerosis that may otherwise be missed with functional testing and invasive angiography.

### High-Risk Plaque

As a majority of acute coronary syndromes (ACS) occur at the site of previously noncalcified and nonobstructive plaque, there has been significant interest in the utility of CTA to detect plaques that may be prone to rupture. In one study, ∼50 % of infarct-related arteries at the time of ACS were non-obstructive on an earlier ICA [43]. Thus, identification of high-risk plaque by non-invasive imaging may help to discriminate patients at increased risk for ACS. While detailed plaque histology cannot be derived from CTA, several plaque features have demonstrated significant association with future ACS, including: the “napkin-ring” sign, spotty calcification, low attenuation plaque, a large necrotic core, and positive remodeling (Fig. 3b–c) [41, 44•, 45]. The prevalence of these features is low, however, with limited sensitivity (∼40 %) and positive predictive value (∼20 %) to predict plaque rupture [41, 46].

### Limitations

Coronary CTA has several limitations that should be considered among implications for management. Importantly, the ability of CTA to distinguish the functional significance of lesions is limited, as approximately half of patients with >50 % stenosis on CTA (and invasive angiography) have no associated ischemia [47•]. Therefore, potential exists for anatomic testing to trigger unnecessary revascularization and increase cost relative to usual care [48]. Additionally, several factors may impair diagnostic image quality (e.g., high heart rate, obesity, and arrhythmias) necessitating further workup. Finally, coronary CTA exposes the patient to contrast and radiation, although hardware and software advances continue to improve image quality while reducing radiation exposure [49].

### Coronary CTA in Asymptomatic Patients

Coronary CTA is not currently recommended in asymptomatic patients, given the lack of demonstrable benefits in screening and low absolute rate of major adverse cardiac events in this population [22••]. In the CONFIRM (COronary CT Angiography EvaluatioN For Clinical Outcomes: An InteRnational Multicenter) registry, the prevalence of obstructive CAD in 7,590 asymptomatic patients was 25 % with a 1.6 % annual mortality rate compared with 0.7 % events/year in patients with no obstructive CAD [50]. Importantly, multivessel CAD (two- or three-vessel/left main) was associated with a higher mortality compared with patients without CAD. Nonetheless, the use of ICA to guide revascularization remains uncertain in asymptomatic patients with multi-vessel and left main obstructive CAD on coronary CTA (Table 2) [52].

**Table 2:** Appropriate use of diagnostic catheterization for suspected CAD on CTA

Coronary CTA	Asymptomatic	Symptomatic
Lesion < 50 % non-left main	Inappropriate	Uncertain
Lesion < 50 % with extensive partly calcified and non-calcified plaque		
Lesion ≥ 50 % non-left main	Uncertain	Appropriate
Lesions ≥ 50 % more than one coronary territory		
Lesion unclear severity, possibly obstructive (non-left main)		
Lesion unclear severity, possibly obstructive (left main)	Appropriate	
Lesion ≥ 50 % left main	Not rated	

*A* appropriate; *I* inappropriate; *U* uncertain, *CAD* coronary artery disease, *CTA* computed tomographic angiography; reproduced with permission from Patel et al. [51••].

As with traditional risk measures, no randomized controlled trials have demonstrated an improvement in outcomes with CTA for screening purposes. In the FACTOR-64 study, 900 asymptomatic patients with diabetes were randomized to aggressive medical therapy ± invasive angiography directed by screening CTA (*n* = 452) versus standard care without CTA (*n* = 448) [53•]. Despite a high prevalence of CAD (23 % with moderate–severe luminal stenosis on coronary CTA; and 41 % with CAC >100), CTA-guided management did not reduce the composite outcome of all-cause mortality, nonfatal MI, or unstable angina at 4 years. However, the authors note that the annual event rate in their study was one-fourth of predicted levels, likely due to outstanding medical management among participants. A majority of patients had well-controlled cardiovascular risk factors at baseline (mean blood pressure = 130/74 mmHg; mean LDL-C = 87 mg/dL, and 74 % statin therapy among study patients), arguing for the importance of medical therapy of high-risk asymptomatic patients and against wide adoption of CTA for screening purposes.

### Symptomatic Patients

Multiple studies have demonstrated a high sensitivity of CTA to detect CAD and prognostic value among low-intermediate patients with stable and acute symptoms concerning for ischemic heart disease [37]. Consequently, CTA is now incorporated among appropriate use criteria [22••] and guideline strategies [9••, 54] for the evaluation of low–intermediate-risk symptomatic patients. Yet, for these prognostic data to result in improved outcomes, patient management and behavior must be impacted and guided in intensity by the test results.

#### Coronary CTA Guides Management and Improves Risk Factors

**Fig. 4:**
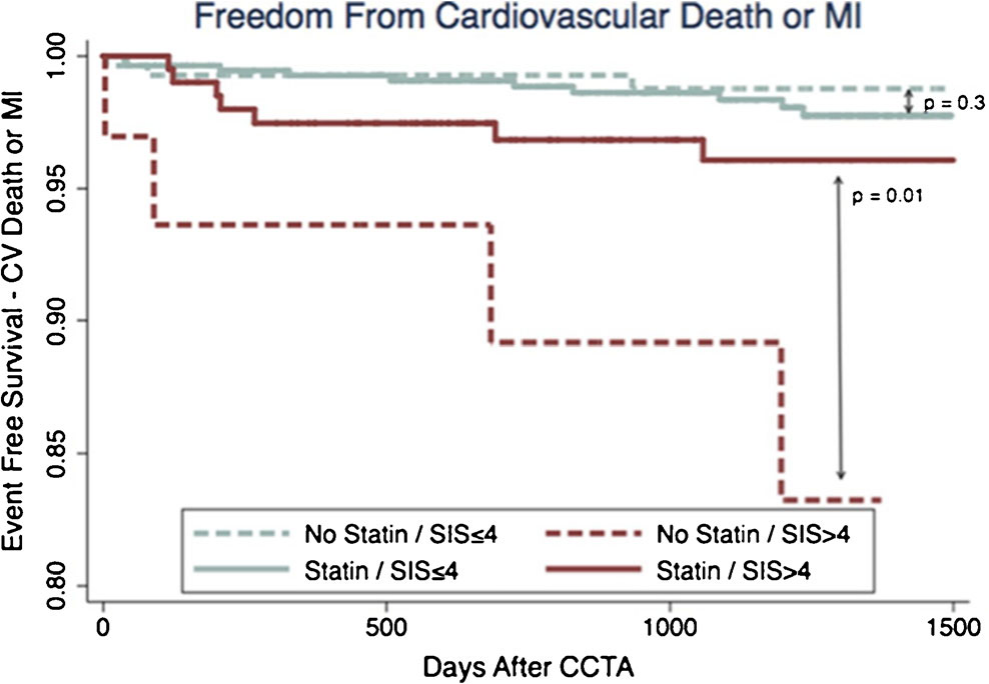
Event-free survival from cardiovascular (CV) death or myocardial infarction (MI) according to presence or absence of statin therapy post-CCTA among those with nonobstructive coronary artery disease (CAD), stratified by extent of disease according to Segment Involvement Score (SIS). Reproduced with permission from Hulten et al. [57•]

**Fig. 5:**
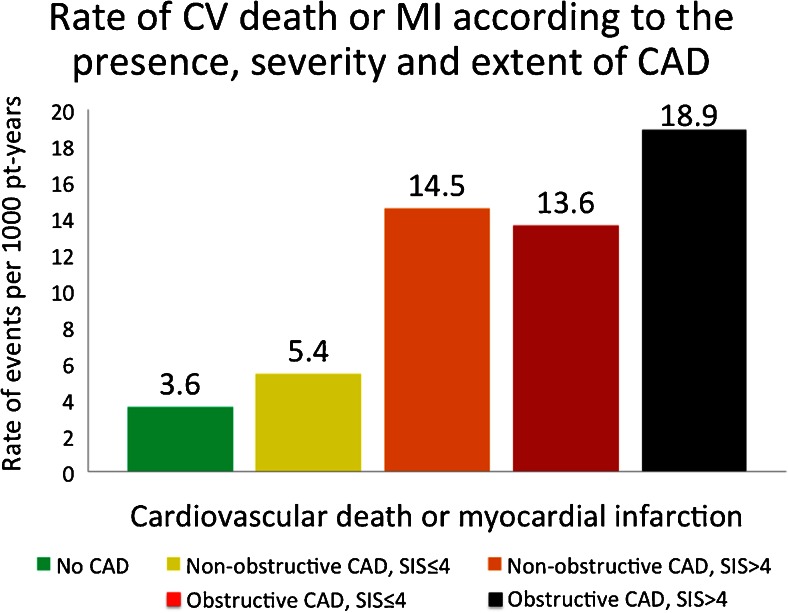
Rate of cardiovascular (CV) death or myocardial infarction (MI) according to the presence, severity, and extent of coronary artery disease (CAD). There is a significant difference (*p* < 0.01) in rates for all comparisons except nonobstructive CAD with segment involvement score (SIS) > 4 and obstructive CAD with SIS ≤ 4. Reproduced with permission from Bittencourt et al. [58]

While no guidelines exist regarding CTA-based treatment strategies, observational data have consistently shown that CTA impacts downstream testing and management, with the potential to guide preventive medical therapies and improve CAD risk factors [55, 56]. Additionally, recent studies have demonstrated an improvement in event-free survival among patients with extensive nonobstructive CAD (SIS > 4) taking statin relative to no statin therapy (Fig. 4) [57•]. Indeed, observational data now suggest that extensive nonobstructive CAD by SIS > 4 carries significant risk for future cardiovascular death or MI, equivalent to patients with obstructive CAD and SIS ≤ 4 (Fig. 5) [58].

Still, significant under-treatment remains among patients with high-risk imaging findings. The Study of myocardial Perfusion and coronary Anatomy imaging Roles in Coronary artery disease (SPARC) trial followed over 1,700 patients following single-photon emission computed tomography, positron emission tomography, or coronary CTA and found that up to 44 % of patients with the highest risk findings went untreated with statins, aspirin, or beta-blockers [59•]. Though the reasons for this remain unclear, potential explanations include a lack of standardized treatment recommendations based on image findings, variability in report interpretation and communication with providers, or variability in patient access and adherence to therapies.

#### Plaque Composition and the Influence of Statin Therapy

In addition to lowering of low-density lipoprotein cholesterol levels, data suggest that statins provide multiple pleiotropic effects leading to reduction in endothelial inflammation and the promotion of plaque stabilization [60, 61]. Additionally, evidence suggests that statin therapy has a significant effect on plaque composition and may alter the thickness of the fibrous cap and stabilize high risk thin cap fibroatheroma [62].

In the CONFIRM registry, an increase in the prevalence of calcified and partially calcified plaque was noted with a decrease in non-calcified plaque among nearly 2,500 patients treated with statins compared with over 4,200 patients in the no-statin arm [63]. The authors postulated that this difference could represent plaque stabilization via conversion from non-calcified to calcified plaque.

#### Management of Coronary CTA Findings

##### No CAD

**Fig. 6:**
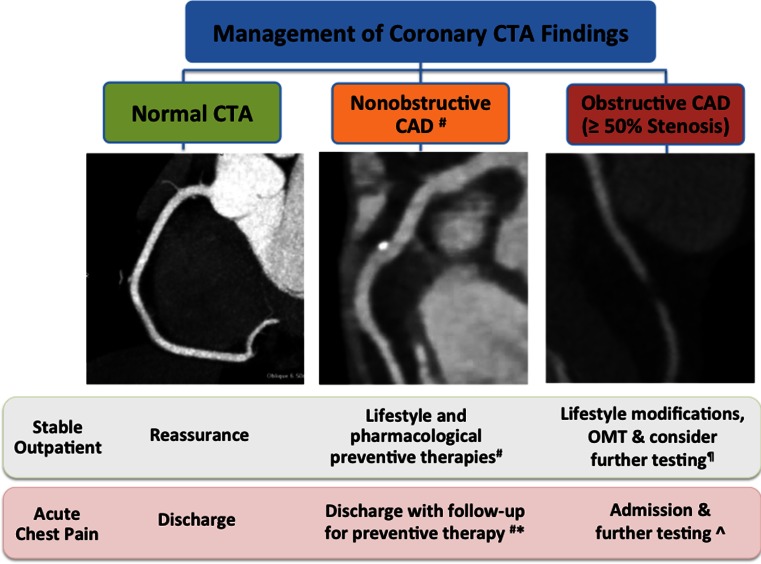
Management of coronary CTA findings. Patients with a normal coronary CTA have a very low (<1 %/year) rate of MACE and can be reassured while ED patients may be safely discharged. In patients with nonobstructive CAD, follow-up lifestyle modification and preventive therapy is recommended. *Number sign* revise risk upwards if diffuse nonobstructive CAD (e.g., segment involvement score > 4) and/or high-risk plaque features present (see Fig. 3). *For acute chest pain, consider repeat biomarkers prior to discharge if extensive nonobstructive CAD present. ^¶^In obstructive CAD with symptoms concerning for stable ischemic heart disease, first-line optimal medical therapy is recommended with consideration for further testing according to appropriate use criteria for testing after CTA (see Table 3) [65••]. **^**In patients with acute chest pain and obstructive CAD, follow guidelines for the diagnosis and management of unstable angina/non-ST-segment elevation myocardial infarction to guide an early invasive versus conservative strategy [66]. *CAD*, coronary artery disease; *CTA*, computed tomographic angiography; *OMT*, optimal medical therapy

Across studies, the absence of CAD on coronary CTA is associated with very favorable prognosis with major adverse cardiac event (MACE) rates of < 1 % out to 7 years [37, 64]. In the majority of cases, patients can be provided with reassurance without further cardiac testing (Fig. 6).

##### Nonobstructive CAD

As presented, extensive non-obstructive CAD (SIS > 4) is associated with a significant risk of future cardiovascular events equivalent to obstructive CAD and SIS < 4 [58]. Furthermore, observational data suggest lower mortality in patients with diffuse nonobstructive CAD managed with statins relative to no statin [67]. Consequently, when CTA identifies diffuse nonobstructive CAD (greater than four coronary segments involved), we recommend a revision of risk upwards with escalation of preventive therapies to include statin use (Fig. 3). Based on an increased risk for ACS among patients with high-risk plaque features, use/intensification of statin therapy appears warranted in this setting. Currently, however, no evidence exists to justify targeted revascularization of lesions on the basis of high-risk plaque features alone.

To guide further management, appropriate use criteria (AUC) for ICA and downstream testing are available following CTA (Tables 2 and 3) [51••, 65••]. With the exception of new/worsening symptoms or left main obstructive CAD, further testing is rarely warranted in patients with nonobstructive CAD unless there is uncertainty in the severity of CTA findings.

**Table 3:** Follow-up testing after CTA when obstructive CAD remains a concern *(Detection and Risk Assessment of Stable Ischemic Heart Disease)*

		Exercise ECG	Stress RNI	Stress echo	Stress MRI	ICA
Sequential testing ≤ 90 days after CTA						
Coronary stenosis or anatomic abnormality of unclear significance on CTA		M	A	A	A	A
Follow-up testing (>90 days): asymptomatic (without ischemic equivalent) or stable symptoms						
Non-obstructive CAD	Low global CAD risk	R	R	R	R	R
	Intermediate/high global CAD risk^a^, CTA < 2 years ago	R	R	R	R	R
	Intermediate/high global CAD risk ^a^, CTA ≥ 2 years ago	M	M	M	M	R
Obstructive CAD	CTA < 2 years ago	R	R	R	R	R
	CTA ≥ 2 years ago	M	M	M	M	R
Follow-up testing: new or worsening symptoms						
Non-obstructive CAD on prior CTA		M	A	A	A	M
Obstructive CAD on prior CTA		M	A	A	A	A

*A* Appropriate; *M* = may be appropriate, *R* rarely appropriate, CAD coronary artery disease, *CTA* computed tomographic angiography, *MRI* magnetic resonance imaging, *ECG* electrocardiogram, *Echo* echocardiography, *RNI* radionuclide imaging

^a^Global CAD risk defined by Adult Treatment Panel III, Prospective Cardiovascular Munster (PROCAM) or similar guidelines [65••]. Reproduced with permission from Wolk et al. [65••]

##### Obstructive CAD

The prognostic value of coronary CTA in patients with obstructive CAD is well documented [37]. In a multivariable analysis of CONFIRM, for example, a significant increase in MACE was observed among patients with obstructive CAD (HR 11.21, *p* < 0.001) when compared with patients with normal CTA [68]. A risk-adjusted dose–response increase in MACE based on the number of vessels with stenosis ≥ 50 % was also demonstrated. Importantly, while many studies examining the prognostic value of CTA report composite MACE including revascularization as an endpoint, significant data have now demonstrated increased risk for hard events (MI/death) in patients with CTA-identified obstructive CAD [37, 58].

In patients with obstructive CAD and symptoms concerning for stable ischemic heart disease, first-line optimal medical therapy is recommended (Fig. 6). Additionally, AUC provide recommendations for further testing when the results would significantly affect individual patient management decisions (Tables 2and 3) [51••, 65••]. As presented, nearly half of patients with obstructive CAD on coronary CTA will have no demonstrable ischemia on stress perfusion imaging [47•]. In these patients, noninvasive functional imaging is generally preferred to better identify patients who would most benefit from ICA/revascularization versus patients with no or minimal ischemia who can be managed with optimal medical therapy.

#### Management of Acute Chest Pain

Multiple randomized controlled trials have demonstrated the ability of coronary CTA to exclude obstructive CAD as a cause for acute chest pain in low–intermediate-risk patients presenting to the emergency department [69]. Thus, patients with no CAD (no plaque or stenosis) may be reassured and discharged without further testing.

While patients with nonobstructive CAD have a very low risk of adverse cardiac events following discharge, a small risk of ACS remains, despite the absence of significant stenosis [70]. In Rule Out Myocardial Infarction Using Computer-Assisted Tomography (ROMICAT) I trial, for example, 6 % of patients with non-obstructive CAD on a blinded coronary CTA were ultimately categorized as having ACS, including three patients with myocardial infarction who were recognized by checking a second set of cardiac biomarkers [71]. Subsequently, among ED patients randomized to coronary CTA in the follow-up multicenter ROMICAT II trial, CTA-identified high-risk plaque features increased the likelihood of ACS independent of obstructive CAD and clinical risk assessment (OR 8.9, *p* = 0.006) [72]. Consequently, in patients with diffuse nonobstructive CAD (SIS > 4) and/or high-risk plaque features, we recommend checking a second set of cardiac biomarkers prior to discharge (Fig. 6). If negative, these patients can be safely discharged with follow-up to guide preventive therapies.

In patients with CTA-identified obstructive CAD, admission and further testing is recommended to guide management. Given concerns regarding the potential for CTA to trigger unnecessary ICA and revascularization, noninvasive functional imaging is generally preferred in low risk, stable patients to determine the functional significance of potentially obstructive CAD. Conversely, in patients with obstructive CAD and concern for unstable angina, consider an early invasive versus conservative management strategy according to recommendations for the management of unstable angina/non-ST-elevation myocardial infarction [66].

#### Anatomy Versus Ischemia to Guide Revascularization

Urgent revascularization guided by ICA for patients presenting with ACS has been well established and supported in current guidelines [73]. In patients with stable ischemic heart disease, however, controversy remains regarding the use of anatomy versus functional testing to guide revascularization. In these patients, revascularization is generally guided by uncontrolled symptoms despite optimal therapy, with notable exception in those with left main CAD [74]. While data suggest patients with diabetes and multi-vessel CAD involving the proximal left anterior descending derive a mortality benefit from revascularization with CABG, controversy remains whether an anatomic approach alone can improve outcomes [75]. Indeed, no survival advantage was seen in either the BARI-2D or COURAGE trials among patients with obstructive CAD undergoing revascularization compared with medical therapy alone [76, 77]. In a post hoc analysis of the COURAGE trial, however, reduced rates of death and myocardial infarction were observed in patients with a reduction in ischemia by ≥5 % among the subset of patients with serial SPECT imaging [78]. Additionally, randomized trials examining FFR to guide revascularization have demonstrated improved cardiovascular outcomes and decreased mortality with cost-savings relative to ICA alone [79, 80].

To date, limited data exist to support CTA-guided revascularization to improve outcomes. In a large, retrospective analysis from CONFIRM, the observed mortality rate for patients with high-risk CAD was significantly lower among patients undergoing revascularization versus medical therapy (2.3 % versus 5.3 %, *p* = 0.008) [52]. Conversely, patients *without* high-risk CAD—defined as left main or multi-vessel obstructive CAD involving the proximal LAD—had higher mortality with revascularization versus medical therapy alone (2.3 % versus 1.0 %, *p* = 0.014). In a separate analysis of CONFIRM, a trend towards reduced mortality was noted in patients with CTA-identified obstructive CAD referred to invasive angiography (HR 0.63, 95 % CI 0.36–1.10, *p* = 0.11) [81]. While these data are inherently limited by their observational design, two large prospective trials are underway to examine the relative benefits of anatomic versus functional testing. The PROMISE trial, which recently closed enrollment, randomized patients with stable symptoms concerning for CAD to an upfront anatomic assessment with coronary CTA compared with functional testing at the discretion of the treating provider [82]. The ISCHEMIA trial, which is still enrolling patients internationally, is randomizing patients with moderate to severe ischemia to optimal medal therapy versus revascularization + optimal medical therapy [83]. All patients in this trial undergo an initial, blinded coronary CTA to exclude patients with no CAD or obstructive left main disease.

#### Future Directions: Value-Based Health Care

Medical imaging has been a particular driving force in the doubling of medical costs since 2000 to over $14 billion annually [84]. This increase in expenditure, as tracked by the Centers for Medicare and Medicaid Services, has doubled the growth in any other healthcare field [84]. Consequently, adoption of “value-based” health care now calls for imaging modalities to satisfy three criteria: high diagnostic accuracy, low resource consumption and cost, and improved clinical outcomes [84]. Despite its proven diagnostic accuracy, the high sensitivity of CTA to detect CAD has raised concerns with respect to test layering and cost.

While studies have consistently demonstrated that CTA decreases hospital admissions and ED costs relative to standard care, concern remains that CTA increases total costs by triggering ICA and revascularization [69]. In a meta-analysis of randomized controlled trials in ED patients, for example, CTA patients had higher rates of ICA (OR 1.36, 95 % CI 1.03–1.80, *p* = 0.030) and subsequent revascularization (OR 1.81, 95 % CI 1.20–2.72, *p* = 0.004) compared with usual care [69]. In a follow-up study, Hulten et al. demonstrated that CAD prevalence is an important driver of downstream resource utilization and cost [48]. Examining ROMICAT I data, the authors found that, when the prevalence of (or inability to exclude) obstructive CAD was less than 30 %, evaluation using coronary CTA was overall cost savings relative to usual care.

In an aging population, the prevalence of atherosclerosis will increase resulting in the potential for increasing numbers of detected coronary lesions of uncertain significance. In a large analysis of Medicare recipients, patients undergoing coronary CTA were more likely to undergo ICA (OR 2.19, *p* < 0.001), PCI (OR 2.49, *p* < 0.001), and CABG (OR 3.00, *p* < 0.001) when compared with patients undergoing SPECT imaging [85]. This resulted in higher CAD-related costs with no difference in mortality among CTA patients compared to SPECT (OR 1.11, *p* = 0.32). Interestingly, however, the authors demonstrated a decreased likelihood of hospitalization for acute MI among CTA patients compared with SPECT (OR = 0.60, *p* = 0.04). A main criticism of this analysis is that the prevalence of CAD in the study population was not reported, and the median age was over 73 years in this Medicare cohort. By comparison, studies examining low–intermediate risk patients considered appropriate candidates for coronary CTA tend to be considerably younger (mean age ∼55 years in CONFIRM) [22••]. In conclusion, the cost effectiveness of CTA strongly depends on optimal patient selection.

## Conclusion

Coronary artery calcium testing and coronary CTA are now well-established modalities with significant evidence supporting their use to guide risk classification and patient management. Among asymptomatic patients without known atherosclerotic cardiovascular disease, CAC testing has been shown to be consistently superior to standard risk scores for predicting cardiovascular risk. Consequently, CAC use is now incorporated among several guidelines and appropriate use criteria for individual risk assessment. Recent studies now support the use of statin therapy in patients with CAC > 0, and individualization of aspirin considering patient ASCVD risk factors, CAC severity, and factors that predispose to bleeding. In symptomatic patients with stable and acute chest pain, coronary CTA provides a high negative predictive value to exclude obstructive CAD. Moreover, the presence, extent and severity of CAD assessed by CTA have been associated with more intensive management and control of ASCVD risk factors with the potential to improve outcomes. Despite this progress, significant under-treatment of patients who are identified as having CAD remains a key area for improvement among patient engagement in lifestyle changes, which remain the foundation of risk prevention. In patients with concern for obstructive CAD, further testing is appropriate when the results of testing are likely to change management, particularly in patients with new or worsening symptoms despite optimal medical therapy. Still, further research is needed to understand long-term cost-effectiveness and downstream impact of CAC and CTA testing among existing strategies, while lowering healthcare costs in an era of value-based care.
